# A Case of Leptospirosis Presenting With Thrombocytopenia, Acute Liver Failure and Acute Renal Failure

**DOI:** 10.7759/cureus.90834

**Published:** 2025-08-23

**Authors:** Sneha Dutta, Mohammad Islam

**Affiliations:** 1 Acute Medicine, Tameside General Hospital, Manchester, GBR

**Keywords:** acute liver failure, acute renal failure, atrial fibrillation, hemofiltration, intensive care, leptospirosis, thrombocytopenia, weil’s disease, zoonotic infections

## Abstract

Leptospirosis is a zoonotic infection caused by the spirochaete *Leptospira interrogans*. Humans usually acquire the organism through contact with contaminated material via mucous membranes or intact skin, although entry is facilitated by skin breaks, such as cuts or abrasions. A vast majority of leptospiral infections are self-limiting; however, in Weil’s disease, which is a more severe form of leptospirosis, patients present with symptoms of jaundice, haemorrhage and renal failure. Arguably, prompt identification and diagnosis of leptospiral infections would reduce mortality and improve patient outcomes. This case report covers a 65-year-old male builder with no significant past medical history, residing in a rodent-infested property, who presented with a seven-day history of flu-like symptoms, jaundice, altered mental status, anuria and coffee-ground vomiting. Laboratory investigations revealed thrombocytopenia, acute kidney injury and acute liver injury, consistent with multi-organ dysfunction. Initial clinical suspicion of leptospirosis was supported by collateral history indicating his dog had recently died with jaundice-like symptoms. A diagnosis of leptospirosis was later confirmed by both a positive polymerase chain reaction (PCR) test and a leptospira antibody test. The patient was managed with intravenous antibiotics, renal support via hemofiltration and mechanical ventilation. He achieved full recovery with subsequent normalisation of liver and renal function.

## Introduction

Leptospirosis is a bacterial infection caused by *Leptospira* species, of which more than 200 have been identified [1]. The four species most often infecting humans are *Leptospira interrogans* Icterohaemorrhagiae, *Leptospira interrogans* Canicola, *Leptospira interrogans* Hardjo and *Leptospira interrogans* Pomona [1,2]. Transmission occurs through contact with urine from infected animals, usually via skin breaks or mucous membranes and can also occur through ingestion of contaminated water [3].

The disease is mainly occupational, affecting farmers, veterinarians and others who work with animals, but it is also seen in tropical regions and among people involved in freshwater activities [4]. Many infections are mild or unrecognised, but severe leptospirosis can cause multi-organ failure and death. One severe form, known as Weil’s disease, is characterised by renal failure, hepatomegaly with liver dysfunction and altered consciousness, often in patients with a history of animal exposure [5]. In such cases, haemodynamic changes resemble sepsis, and acute kidney injury (AKI) is caused by both the direct nephrotoxic effect of *Leptospira* and toxin-induced immune responses [6]. Because its early clinical features are nonspecific and overlap with sepsis, viral infections and vasculitis, diagnosis can be difficult and often requires systematic exclusion of alternative causes.

This report presents an adult with no relevant travel history who developed reduced Glasgow Coma Scale (GCS), thrombocytopenia and combined hepatic and renal failure. A wide differential was excluded, but collateral exposure history was the key to raising suspicion for leptospirosis, which was later confirmed by polymerase chain reaction (PCR) and leptospira antibody tests. Early recognition, treatment with ceftriaxone, and organ support led to full recovery, highlighting the importance of exposure history in guiding diagnosis and management.

## Case presentation

A 65-year-old gentleman presented feeling generally unwell and confused. He described a seven-day history of flu-like symptoms, including fever, malaise, headaches, myalgia and worsening confusion. Over the preceding 24 hours, he had developed yellow discolouration of the eyes and had one episode of coffee-ground vomiting. He had no past medical history, took no regular medications and had no known drug allergies. The patient worked as a builder and smoked about 20 cigarettes per day. He reported daily alcohol consumption, amounting to approximately four to five cans. Despite his high alcohol intake, he had no known history of alcohol-related liver disease and no previous hospital admissions or surgeries.

On admission

On admission, observations were as follows: Respiratory rate of 22 breaths per minute, peripheral capillary oxygen saturation was 99% on room air with an irregular heart rate of 144 beats per minute, the patient was febrile with a temperature reading of 39.2°C and a blood pressure of 107/63mmHg. On general examination, he had a GCS of 14/15; his chest was clear anteriorly, but a tender right upper quadrant abdomen was found.

Initial investigations on admission are shown in Tables 1-2.

**Table 1:** Arterial blood gas on admission cBase: base excess (mmol/L); cCa²⁺: concentration of calcium (mmol/L); cCl-: concentration of chloride (mmol/L); cGlu: glucose (mmol/L); cHCO₃-: bicarbonate (mmol/L); cK⁺: concentration of potassium (mmol/L); cLac: concentration of lactate (mmol/L); cNa⁺: concentration of sodium (mmol/L); ctHb: concentration of haemoglobin (g/L); pCO₂: partial pressure of carbon dioxide (kPa); pH: arterial blood pH; pO₂: partial pressure of oxygen (kPa); SO₂: arterial oxygen saturation (%). “-” indicates no reference range.

	Values	Reference range
pH	7.13	7.340-7.450
pCO_2_	5.01	4.50-6.10 (kPa)
pO_2_	35.9	10.0-14.0 (kPa)
ctHb	175	117-174 (g/L)
sO_2_	99.4	95.0-99.0 (%)
cK^+^	4.3	3.5-5.3 (mmol/L)
cNa^+^	137	135-146 (mmol/L)
cCa^2+^	1.04	1.13-1.32 (mmol/L)
cCl^-^	97	95-108 (mmol/L)
cGlu	6.7	2.6-7.8 (mmol/L)
cLac	1.4	0.5-2.5 (mmol/L)
cBase	-6.6	-
cHCO₃-	19.2	-

In Table 1, the arterial blood gas (ABG) results point to metabolic acidosis. 

**Table 2:** Initial investigations on admission Albumin: serum albumin concentration (g/L); ALP: alkaline phosphatase, marker of cholestasis or bone turnover (U/L); ALT: alanine aminotransferase, marker of hepatocellular injury (U/L); Amylase: serum amylase, marker of pancreatic disease (U/L); Bilirubin: total serum bilirubin (µmol/L); Creatinine: serum creatinine (µmol/L); CRP: C-reactive protein, inflammatory marker (mg/L); eGFR: estimated glomerular filtration rate (mL/minute/1.73 m²); GGT: gamma-glutamyl transferase, marker of biliary and hepatocellular disease (U/L); Haemoglobin level: concentration of haemoglobin in blood (g/L); INR: international normalised ratio, measure of blood clotting (ratio); K⁺: serum potassium (mmol/L); MCV: mean cell volume, average size of red blood cells (fL); Na⁺: serum sodium (mmol/L); Neutrophils: absolute neutrophil count (×10⁹/L); Platelet: platelet count (×10⁹/L); Urea: blood urea concentration (mmol/L); WBC: white blood cell count (×10⁹/L).

Investigation	Values	Reference range
Haemoglobin level	172	115-165 (g/L)
WBC	15.2	4.5-11.0 (×10^9^/L)
Neutrophils	12.8	2.0-7.5 (×10^9^/L)
Platelet	13	150-450 (×10^9^/L)
MCV	97	80-100 (fL)
INR	1.0	0.8-1.2
Na^+^	Haemolysed	135-145 (mmol/L)
K^+^	Haemolysed	3.5-5.3 (mmol/L)
Urea	54.2	2.5-7.8 (mmol/L)
Creatinine	906	54-110 (µmol/L)
eGFR	5	>90 (mL/minute/1.73 m^2^)
ALT	218	<60 (U/L)
ALP	166	30-130 (U/L)
Bilirubin	368	0-21 (µmol/L)
Albumin	28.3	31.0-45.0 (g/L)
GGT	44	15-40 (U/L)
CRP	189	0-5 (mg/L)
Amylase	127	30-118 (U/L)

The next set of investigations, as shown in Table 2, reveals evidence of thrombocytopenia, AKI, and acute liver injury.

**Figure 1:**
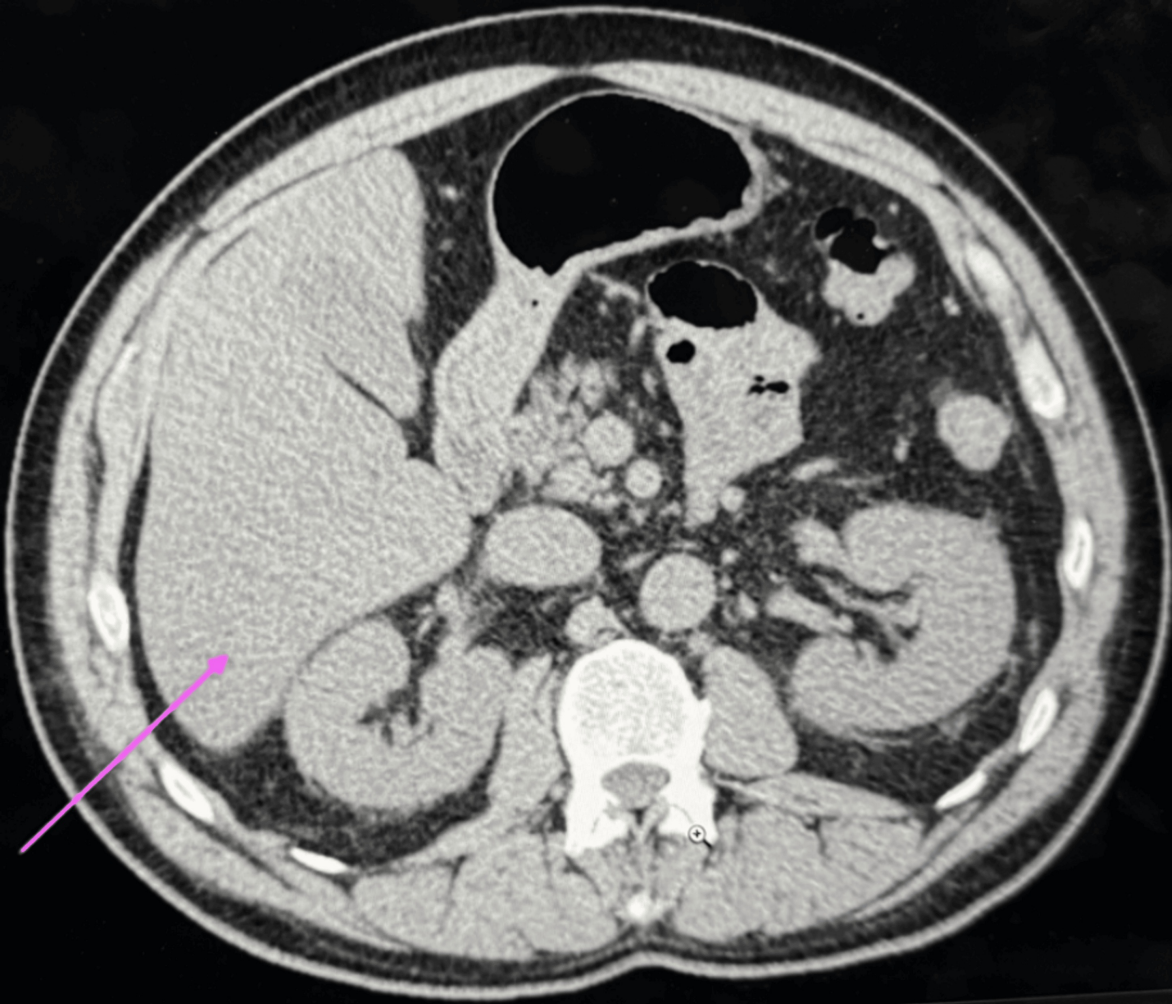
Computed tomography of thorax, abdomen, and pelvis for patient showing no biliary obstruction Pink arrow indicates that the liver appears normal.

Imaging and further tests were also performed. A computed tomography (CT) scan of the thorax, abdomen, and pelvis (CT TAP) showed no biliary obstruction or malignancy, with only minor lung base inflammation. A CT scan of the head was normal. The COVID-19 reverse transcriptase-polymerase chain reaction (RT-PCR) test was negative (Figure 1).

**Figure 2:**
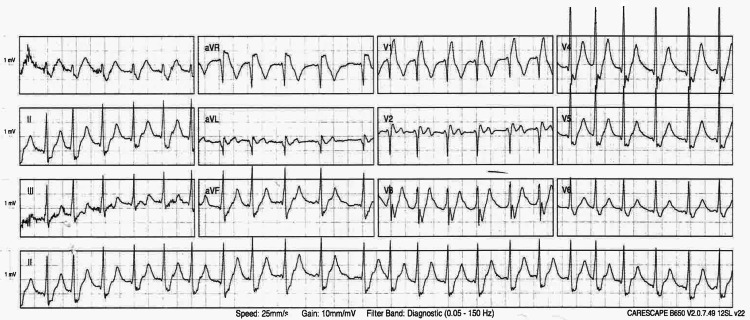
Electrocardiogram (ECG) showing atrial fibrillation with fast ventricular rate Mean heart rate: 150 bpm; rhythm: irregular; QRS axis: greater than +90°; atrial fibrillation with rapid ventricular response; right bundle branch block.

An ECG was also carried out, as shown in Figure 2.

The patient’s ECG (Figure 2) demonstrated new atrial fibrillation (AF).

Initial management

A total of 2.5 L of intravenous (IV) fluids was administered over eight hours. The patient also received bisoprolol 5 mg for fast AF, ondansetron 4 mg, IV proton pump inhibitor (PPI) 40 mg stat, and empirical IV cefuroxime 1.5 g (renal dose) for suspected sepsis.

24-hour post-admission

The patient was hypotensive (BP 68/45 mmHg), tachycardic (HR 114 bpm) and was noticed to be anuric (less than 50 mL/24 hours), needing intensive care review and transfer. His GCS deteriorated to 8/15, and he was unable to maintain his airway and ventilation, which necessitated intubation after sedation with propofol. In addition to this, he was treated with IV noradrenaline infusion at 16 mL/hour (double strength: 160 mcg/mL), equivalent to 0.61 mcg/kg/minute, titrated between 0-20 mL/hour (0-0.76 mcg/kg/minute) for inotropic support, with close monitoring of fluid balance.

Other treatments included IV amiodarone for persistent AF (300 mg bolus, then approximately 900 mg over 24 hours) while on noradrenaline, and IV N-acetylcysteine per the local acute liver failure protocol (50 mg/kg in 500 mL over four hours, then 100 mg/kg over 24 hours for three days) to replenish glutathione and mitigate oxidative injury. IV piperacillin-tazobactam was started as empiric cover 4.5 g IV stat, then 4.5 g IV tds (three times a day) (every eight hours) and hemofiltration to combat his anuric state.

Day 2 intensive therapy unit (ITU)

On day 2, the patient remained anuric, intubated and supported with ionotropic agents. His case was discussed with the local microbiology team who advised to stop piperacillin-tazobactam and start him on IV ceftriaxone 2 g twice daily and aciclovir 750 mg tds to cover for viral encephalitis, as a lumbar puncture was not possible considering his very low platelet count.

His case was also discussed with the nephrology team, who recommended further investigations, including vasculitis screen, lactate dehydrogenase (LDH), blood film, extractable nuclear antigen (ENA) antibodies (Ab), anti-neutrophil cytoplasmic antibodies (ANCA), and anti-glomerular basement membrane (anti-GBM) antibodies Ab. The haematology team was consulted regarding his presentations, signs and symptoms. They requested a blood film and determined that his case was unlikely to be explained by disseminated intravascular coagulopathy or thrombotic thrombocytopenic purpura.

The local gastroenterology team was also called to review his coffee-ground vomiting and tender right upper quadrant. Their opinion was that the case was unlikely to be explained by the onset of acute liver disease. On further questioning of his family relatives to get collateral history, it was discovered that his house was heavily infested with rats, and his pet dog had died recently from a strange illness. The illness also caused yellowish colouration of the dog's eyes. Based on these findings, a working diagnosis of Weil’s disease was made, and blood samples were sent for *Leptospira* IgM antibody and PCR testing.

Day 3 ITU

He remained anuric, so hemofiltration was continued. He was still intubated and still needed ionotropic support, but his amiodarone maintenance was stopped as his AF had resolved. The patient's blood culture and urine culture showed no growth of any organism.

Day 4 ITU

On day 4, N-acetylcysteine therapy was stopped. Urine output improved to 0-40 mL/hour; however, hemofiltration was continued, and the patient still remained intubated, ventilated and supported using ionotropic agents.

Further investigations

Further investigations were conducted to systematically rule out any alternative underlying pathology and ensure a comprehensive evaluation of the patient’s condition. The findings are presented in Tables 3-6.

**Table 3:** Further investigations ANA: antinuclear antigen; CK: creatine kinase; CRP: C-reactive protein; DAT: direct antiglobulin test; ENA: extractable nuclear antigen; ESR: erythrocyte sedimentation rate; LDH: lactate dehydrogenase; PSA: prostate-specific antigen; TIBC: total iron-binding capacity; TSH: thyroid-stimulating hormone. “-” indicates no reference range applicable.

Investigation	Value	Reference range
Ammonia	96	<50.0 (µmol/L)
LDH	1507	350-600 (IU/L)
ESR	39	<14 (mm/hour)
CK	2976	40-320 (U/L)
PSA	2.2	<3.9 (µg/L)
Urate	99	200-430 (µmol/L)
Amylase	127	29-103 (U/L)
Troponin	252.2	<19.8 (ng/L)
Ferritin	245	24-337 (ug/L)
Iron	16.3	13-32 (µmol/L)
Transferrin	1.6	2.0-3.6 (g/L)
TIBC	40	45-70 (µmol/L)
D-Dimer	4634	0-500 (µg/L)
DAT	Negative	-
Haptoglobin	31	36-195 (mg/dL)
Conjugated bilirubin	425	0.0-3.5 (µmol/L)
ENA	<0.2	<0.9 (AI)
Vitamin B12	450	145-910 (ng/L)
TSH	0.41	0.40-4.50 (mU/L)
Free T4	12.7	7.0-17.0 (pmol/L)
ANA	Normal	-
CRP	189	0-8 (mg/L)
HIV	Negative	-
Salicylate level	<10	-
Paracetamol level	<10	-
Immunoglobulin	Normal	-

**Table 4:** Patient clotting profile through admission APTT: activated partial thromboplastin time; INR: international normalised ratio; "-": no value available.

Date	Platelet (150-450 × 10^9^/L)	INR (0.8-1.2)	APTT (25-35 seconds)	APTT ratio (0.8-1.2)	Fibrinogen (1.9-8.0 g/L)
30/10/21	16	1.1	25.8	1	6.3
31/10/21	48	1.1	28.8	1.1	6.6
1/11/21	26	1.2	24	1	6.1
2/11/21	20	1.1	24.6	O.9	6.3
3/11/21	25	0.9	21.6	0.8	6.1
17/11/21	400	-	-	-	-
24/11/21	370	-	-	-	-

**Table 5:** Renal function tests from admission to recovery (including two weeks of renal support) Dates were omitted for conciseness.

Date	Na+ (133-146 mmol/L)	K+ (3.5-5.5 mmol/L)	Urea (2.5-7.8 mmol/L)	Creatinine (54-110 µmol/L)	eGFR (>90 mL/minute/1.73 m^2^)
30/10/21	N/A	N/A	54.2	906	5.1
31/10/21	142	5.1	66.8	933	5.0
01/11/21 (haemofiltration)	138	4.7	21.4	393	15.3
02/11/21 (haemofiltration)	138	4.3	15.1	297	18.8
03/11/21 (haemofiltration)	136	4.0	10.9	244	23.8
16/11/21	143	3.8	38.7	538	9.4
19/11/21	142	3.6	27.7	404	13.1
24/11/21	140	3.8	13.5	218	27.2
24/12/21	139	4.9	4.6	97	>90

**Table 6:** Liver function tests from admission to recovery ALP: alkaline phosphatase; ALT: alanine aminotransferase; "-": values were not available. Dates were omitted for conciseness.

Date	ALT (<60 U/L)	ALP (30-130 U/L)	Bilirubin (0-21 µmol/L)	GGT (15-40 U/L)	Albumin (31-45 g/L)
30/10/21	218	164	368	44	28.3
31/10/21	168	135	472	27	25
1/11/21	139	-	420	-	-
2/11/21	100	-	387	-	-
3/11/21	97	140	329	-	15.1
16/11/21	54	209	76	27	-
19/11/21	54	156	70	66	-
22/11/21	35	147	66	78	-
24/12/21	17	34	27	106	-

The patient’s blood film was also tested, with the following results: WBC showed leukocytosis with neutrophilia; RBC showed occasional target cells with no schistocytosis; and platelets showed thrombocytopenia with giant platelets.

The comments by the haematologist on these results were as follows: the platelet count appeared genuine, with no platelet clumps, and occasional large platelets were seen. The WBCs showed leukocytosis with neutrophilia and occasional myelocytes, and the neutrophils demonstrated toxic granulation and vacuolation. The RBCs showed occasional target cells with no significant schistocytosis, while the platelets showed thrombocytopenia with giant platelets. In conclusion, the findings were consistent with severe sepsis, with no evidence of microangiopathic haemolytic anaemia, and the leucoerythroblastic blood picture suggested a stressed marrow.

The clinical presentation of the patient, in conjunction with the findings from Tables 2-3 as well as the peripheral blood film analysis, is highly suggestive of sepsis with multi-organ involvement.

The results of Tables 3-4 and the peripheral blood film showed leucocytosis with neutrophilia and toxic granulation and vacuolation, occasional myelocytes, thrombocytopenia with giant platelets, and occasional target cells; no schistocytes were seen. Overall, the findings were consistent with sepsis and suggested a leuco-erythroblastic picture indicative of stressed marrow, with no evidence of micro-angiopathic haemolytic anaemia.

Renal function tests were performed daily since admission, as shown in Table 5. The results indicate that severe AKI improved with two weeks of renal support and subsequently returned to baseline.

Similarly, liver function tests were carried out daily, and the results, shown in Table 6, demonstrated predominant conjugated hyperbilirubinemia. Liver injury was found to be significantly improved, as indicated by the results in Table 6.

Clinical course and outcome

As suspected, the PCR test was positive, and subsequent results confirmed *Leptospira* IgM antibody positivity. IV ceftriaxone of 2 g twice daily was commenced, given for four days at the referring hospital and continued for a further three days at the tertiary care centre, completing a seven-day course in total. At the tertiary centre, renal replacement therapy was discontinued after six days, and the patient was successfully extubated on day 7. In total, he required 14 days in intensive care between the referring hospital and the tertiary centre. Following transfer to a ward, no further antibiotics were administered. He made a gradual clinical improvement, with normalisation of renal and hepatic function, and was discharged home in stable condition.

## Discussion

Leptospirosis is a zoonotic infection with higher incidence in tropical and subtropical regions, though cases are also reported in temperate areas. The World Health Organization (WHO) estimates the annual incidence to range from 0.1 to 1 per 100,000 in temperate regions and over 10 per 100,000 in tropical regions, influenced by environmental and socioeconomic factors [1]. The incubation period is typically one to two weeks [2]. The disease progresses through two phases: an initial septicaemic phase with nonspecific flu-like symptoms, followed by an immune-mediated phase with more severe features such as renal impairment, hepatomegaly, jaundice, and haemorrhagic manifestations [3,4]. The patient in this case presented directly in the immune-mediated phase with hepatorenal dysfunction, thrombocytopenia, and reduced GCS, without recognition of the earlier febrile illness.

Exposure history is essential in diagnosing leptospirosis, particularly in non-endemic areas [2,5]. In this case, collateral information revealed a rodent-infested home and the recent unexplained jaundice-related death of the patient’s dog, which raised suspicion for leptospirosis. The absence of recent travel history also helped to exclude other tropical infections such as malaria and dengue, which may present with fever, jaundice, and thrombocytopenia. These factors supported the decision to test for *Leptospira*.

Laboratory abnormalities commonly reported in leptospirosis include anaemia, leukocytosis and thrombocytopenia [1]. Liver function tests often show elevated transaminases and hyperbilirubinaemia, usually with normal alkaline phosphatase [5]. The findings in this case demonstrated marked thrombocytopenia, conjugated hyperbilirubinaemia, and elevated creatine kinase, with exclusion of microangiopathic haemolysis on blood film, were consistent with severe systemic involvement.

AKI in leptospirosis may result from direct nephrotoxicity, immune-mediated damage, haemodynamic instability, jaundice and rhabdomyolysis [6]. Thrombocytopenia is also frequent, related to platelet consumption, immune-mediated destruction and reduced bone marrow production. It increases bleeding risk and is associated with renal and hepatic dysfunction [7]. Previous case reports describe AKI requiring renal replacement therapy, with incomplete recovery in some patients. In this case, renal replacement therapy was required for two weeks, after which both renal and hepatic function recovered completely.

Diagnostic confirmation relies on a combination of serology and PCR testing. Modified Faine’s criteria have been developed to support clinical diagnosis by incorporating epidemiological, clinical, and basic laboratory features. While it may assist in early recognition and empiric treatment, the performance is variable and confirmatory testing with serology or PCR remains necessary [8]. In this case, PCR and serology tests ultimately confirmed the diagnosis.

Dogs are recognised reservoirs of *Leptospira*, and close contact increases human risk [9]. Human cases have risen in areas with low vaccination coverage. Use of quadrivalent canine vaccines has been shown to reduce incidence, highlighting their importance for public health [10].

This case shows leptospirosis presenting with hepatorenal dysfunction, thrombocytopenia, and reduced GCS in a patient without travel history. Zoonotic exposure provided the diagnostic clue. The presentation mimicked systemic illnesses such as sepsis and vasculitis. Early recognition, antibiotic therapy, and organ support resulted in complete recovery.l

## Conclusions

This case highlights the vital importance of obtaining a detailed exposure history, particularly regarding zoonotic contacts, which raises clinical suspicion for leptospirosis even in non-endemic areas. Normal CT imaging, the absence of microangiopathic haemolysis on blood film, and negative ENA/ANCA/anti-GBM reduced the likelihood of alternative biliary, haematologic, and vasculitic causes. A positive PCR test and leptospira antibody confirmed the diagnosis. Early ceftriaxone, with renal replacement and ventilatory support, was followed by complete hepatic and renal recovery. Prompt recognition based on targeted exposure history, alongside timely antibiotic therapy and supportive care while awaiting confirmatory testing, remains crucial for improving outcomes.
